# Jejunal perforation complicating dermatomyositis

**DOI:** 10.1016/j.ijscr.2019.04.010

**Published:** 2019-04-10

**Authors:** Donghyoun Lee, Woo Seong Jeong, Chang Lim Hyun, Jinseok Kim

**Affiliations:** a Department of Surgery, Jeju National University Hospital, Jeju National University School of Medicine, Jeju Self-governing Province, South Korea; b Division of Rheumatology, Department of Internal Medicine, Jeju National University Hospital, Jeju National University School of Medicine, Jeju Self-governing Province, South Korea; c Department of Pathology, Jeju National University Hospital, Jeju National University School of Medicine, Jeju Self-governing Province, South Korea

**Keywords:** Dermatomyositis, Perforation, Small bowel, Intestinal

## Abstract

**Introduction:**

Small bowel perforation is rare in dermatomyositis (DM). However, it is associated with high rates of morbidity and mortality. In line with the SCARE criteria, we describe a case of jejunal perforation for a DM patient (Agha et al., 2018 [1]).

**Case presentation:**

A 63-year-old woman had been treated for DM with high dose steroid 1 month prior to the onset of severe abdominal pain. Computed tomography (CT) revealed free air in abdominal cavity and fluid around the small bowel. She was diagnosed with small bowel perforation and underwent emergency surgery. Emergent surgery showed perforated jejunum which was resected. Pathologic reports revealed mesenteric small-vessel vasculitis with a perivascular inflammatory cell infiltration.

**Conclusion:**

Perforation of the small bowel for DM patients is rare. However, the early diagnosis of bowel perforation is difficult in DM because it can mimic other gastrointestinal manifestations such as ileus, ischemic colitis and peritonitis. To minimize mortality via an early diagnosis and a timely treatment, it is important to examine the patient’s clinical history and employ a proper medical imaging modality such as CT even when lab findings are nonspecific and atypical.

## Highlights

• Jejunal perforation in dermatomyositis is very rare but fatal.
• The authors describe an unusual case of jejunal perforation due to ischemic change, which is a very rare complication of DM.
• To minimize mortality via an early diagnosis and a timely treatment, it is important to examine clinical history and to employ a proper medical imaging modality such as CT even when lab findings are nonspecific and atypical.

## 1 Introduction

Dermatomyositis (DM) is an autoimmune inflammatory myopathy characterized by proximal myopathy with inflammatory infiltrates in the muscle tissue and characteristic rash (i.e., heliotrope rash or Gottron’s papules). The peak incidence of DM occurs with a bimodal distribution of 5–14 and 45–60 years of age [2]. The incidence rates of DM and polymyositis (PM) are 1.4 and 3.8, while the prevalence rates are 5.8, and 9.7 cases per 100,000 people in the United States, respectively [3]. Prior to the availability of corticosteroids, the prognosis of DM and PM was poor [4]. After using corticosteroid, the 5- and 10-year survival rates for DM increased by 63% and 53%, respectively [5]. DM mainly affects the skin and muscle, and also affects the lung and gastrointestinal (GI) tract. In this case report, the authors describe an unusual case of jejunal perforation due to ischemic change, which is a very rare complication of DM. This presented work has been reported in line with the SCARE (2018) criteria [1,6].

## 2 Case

**Fig. 1:**
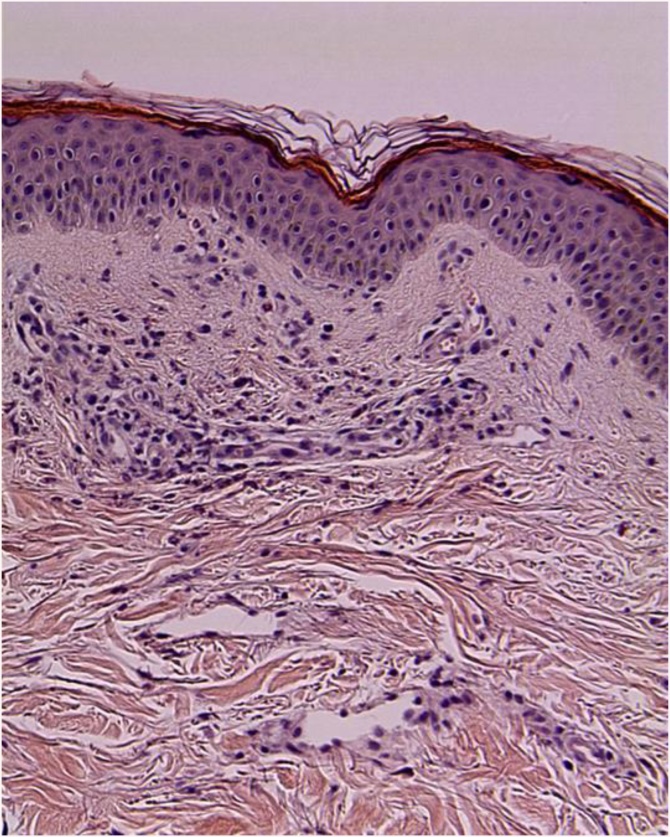
Prominent hyperkeratosis, mucin deposition and upper dermal perivascular mononuclear infiltrates.

**Fig. 2:**
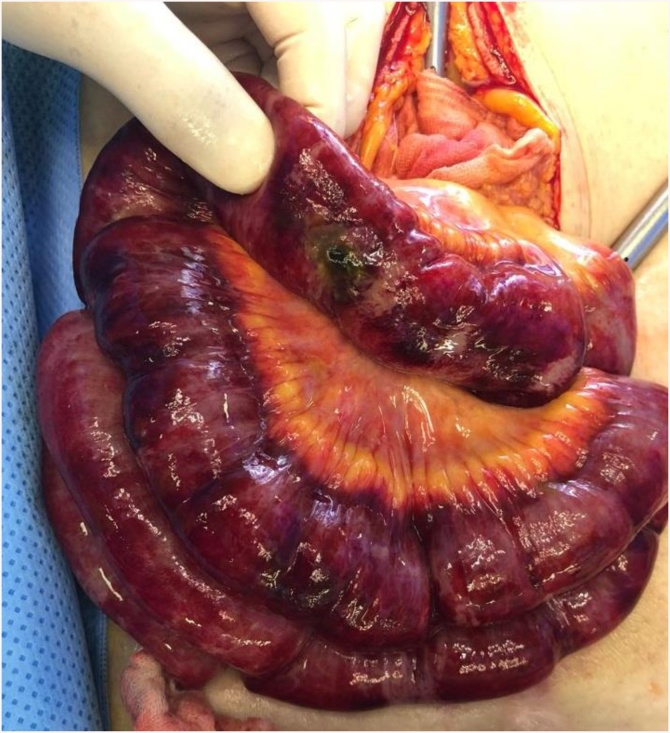
Intra-operative findings: 1.5-cm perforation hole over jejunum. The bowel around the perforation was found to be inflamed and ugly looking. Overall color of bowel and mesentery turned dark.

**Fig. 3:**
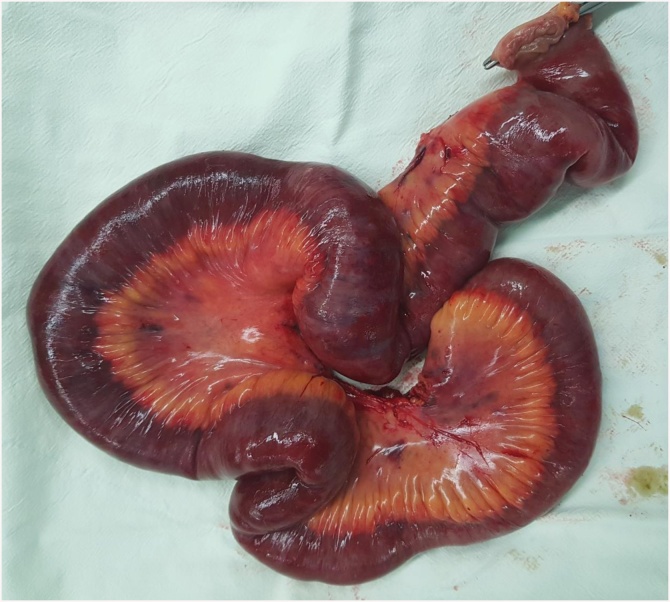
Post-operative findings: Specimen of the resected segment of the small bowel showing massive intestinal and mesenteric infarction.

**Fig. 4:**
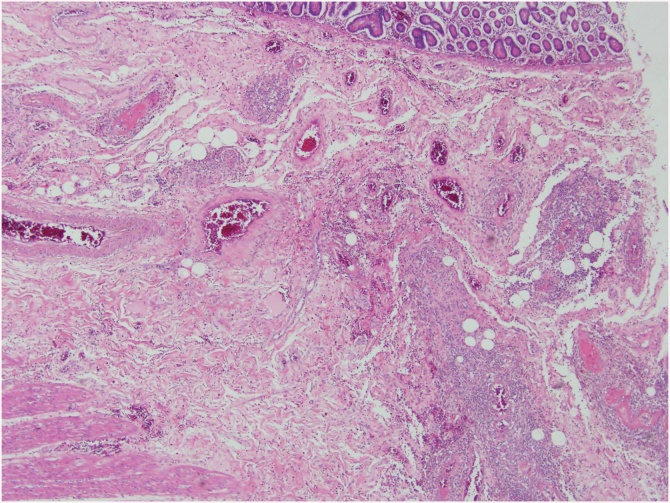

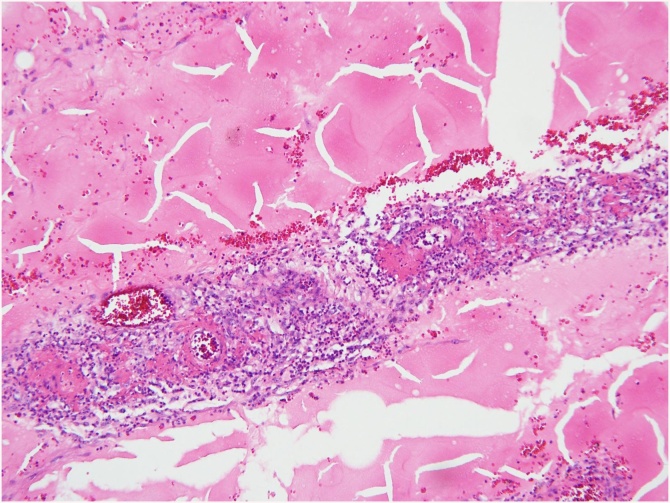
(A) Mesenteric small-vessel vasculitis. (B) Vascular thrombosis with a perivascular inflammatory cell infiltration.

A 63-year-old female patient presented to the Department of Internal Medicine with a fever (>38 °C) that had persisted for two days and lower abdominal pain. The patient was admitted to the hospital due to gait disturbance that started two months earlier. Brain electromyography, brain magnetic resonance imaging, and nerve conduction study failed to reveal the cause of gait disturbance. Positron emission tomography showed mild uptake of fluorodeoxyglucose in the soft tissue around the nose and posterior neck muscle area at the C1 level. Skin biopsy showed hyperkeratosis, mucin deposition and upper dermal perivascular mononuclear infiltrates (Fig. 1). In addition, muscle biopsy showed many perifascicular atrophic myofibers and subsarcolemmal accumulation of swollen mitochondria by electron microscopy. The patient was diagnosed with DM. She was treated with high-dose steroid therapy. Intravenous methylprednisolone (MPD, 50 mg daily) was initiated, but there was no clinical response. Thus, azathioprine (50 mg daily) was added while tapering MPD. In the fourth week of hospitalization, fever above 38 °C persisted and she was diagnosed with pneumonia. She was treated with ceftriaxone (2 g daily) and clindamycin (600 mg every 8 h). The ceftriaxone and clindamycin therapy was replaced after 2 days with meropenem (1 g daily). Although her symptoms subsided gradually over the first week, they recurred thereafter and did not remit despite adding intravenous vancomycin (1 g every 12 h). Three weeks after the pneumonia diagnosis, she complained of sudden onset of abdominal cramping. On physical examination, she was pale and in distress due to continuous abdominal pain. Abdominal CT scans showed a moderate amount of free air and fluid within the upper abdomen. Initial hematology evaluation showed the following: hemoglobin 9.0 g/dL, white blood cells (WBC) 5.7 × 10^3^/μL (absolute neutrophil count 4470/μL), and platelets 50 × 10^3^/μL. She had high-grade fever (39 °C). Although her purse rate was high (118 beats/min), blood pressure (123/80 mmHg) was normal. Following an emergent surgical consultation, the patient was diagnosed with pan-peritonitis due to small bowel perforation and promptly transferred to the Department of Surgery, whereby a diagnostic surgical exploration was performed. A 2 × 1 cm sized perforation in the antimesenteric border of proximal jejunum was identified (Fig. 2). A moderate amount of dirty ascites with small bowel contents was found intraperitoneally, and the surface of jejunum and its mesentery were covered with pus. In addition to the perforation, a 10 cm discoloration was observed on both sides of the perforation site, suggesting an ischemic change. Approximately 50 cm of discolored jejunum, including the perforation, was resected and end-to-end anastomosis was performed (Fig. 3). Upon opening the specimen, the mucosa revealed marked hemorrhage and necrotic change. Representative sections were embedded for histological analysis. On histologic examination, the serosal surface showed hemorrhage and inflammatory exudates with perforation. Pathologic examination showed mesenteric small-vessel vasculitis (Fig. 4-A). In addition, there was vascular thrombosis with a perivascular inflammatory cell infiltration (Fig. 4-B). After surgery, the patient was kept in the intensive care unit. Until the fifth postoperative day, she experienced episodic hypotension and her hemoglobin decreased continuously. Two units of packed red blood cells and 6–7 units of platelets were transfused daily. On the third postoperative day, both blood and sputum cultures revealed *Candida parapsilosis*. Cultures continuously remained positive despite intravenous administration of fluconazole (400 mg daily). Finally, she developed multiorgan failure and expired 11 days after surgery.

## 3 Discussion

DM is a rare, chronic inflammatory disease. It is caused by vasculitis determined by humoral factors with subsequent inflammatory cell accumulation, mainly T CD4+ and B cells, which infiltrate myocytes leading to its vacuolization and degeneration (mainly in the skeletal muscles, rarely in the smooth muscles) [7]. The diagnostic criteria for DM include nine characteristics in total, including characteristic skin lesions, proximal muscle weakness and pain, elevated serum creatine kinase, and positive anti-Jo-1 antibody test. DM can be diagnosed with if four of the eight other criteria are met in addition to skin lesions [8]. The patient reported here had typical skin lesions and met other criteria such as myogenic changes on electromyography, systemic inflammatory signs, and pathologic findings compatible with inflammatory myositis. Hence, the patient was diagnosed with DM.

Although GI manifestations occur in up to 50% of DM patients, most cases show symptoms such as dysphagia, esophageal reflux, esophageal dysmotility, delayed gastric emptying, and decreased intestinal motility [9,10]. DM-induced intestinal perforation is very rare, but steadily reported [11,12]. The authors’ own counting of citations indexed in PubMed suggests that fewer than 10 cases have been reported since 1995. Previous case reports show that GI manifestations of DM can be improved via immunosuppression^10^]. However, the patient in this case was diagnosed with sudden onset perforation despite high-dose steroid therapy. A possible explanation is that perforation resulted from intestinal ischemic change due to severe vasculopathy, which was confirmed by pathologic report.

The pathologic examination of intestinal vessels in this case showed small-vessel vasculitis with intra-vascular thrombosis. We conjecture that there are two factors jointly contributing this vasculopathy. The first point is endothelial injury due to DM itself. The existing studies reported that 44% of patients with DM and PM are found with anti-endothelial antibodies such as IgG and IgM [13]. These anti-endothelial antibodies are known to be closely associated with interstitial lung disease [14,15]. However, no ILD was found in the patient of this case. The second point is intestinal perforation due to steroid therapy. According a recent large-scale meta-analysis examining 159 studies, the use of corticosteroids may increase the odds ratio by 40% for GI bleeding or perforation among hospitalized patents [16]. Due to high-dose steroid therapy (MPD 50 mg daily for 6 weeks), the patient of this case was at an increased risk of GI perforation.

The treatment for spontaneous small bowel perforation of the DM is based on the same principles as other perforations of the intestine. Initial management of small bowel perforation consisted of intravenous fluid therapy, cessation of oral intake and intravenous broad-spectrum antibiotics. Surgical options range from conservative to abdominal exploration including primary repair, enterostomy and segmental resection. Since the first laparoscopic repair of small bowel perforation was performed in 2005, pure laparoscopic or extracorporeal laparoscopic-assisted resection of a perforated small bowel has been widely performed [17]. In this case, a laparoscopic approach was initially attempted; however, due to the large amount of the fecal soiling and wide range of the necrotized small bowel mesentery, it was converted to open surgery.

The key clinical feature of this case is sudden onset abdominal pain with normal WBC count in DM patient. In conclusion, intestinal perforation among DM patients has been extremely rare since the development of steroid therapy. However, despite high-dose steroid therapy, bowel perforation can be caused by ischemic changes due to vasculitis. Therefore, immediate examinations such as CT scans should be conducted when bowel perforation or ischemic change is suspected. We argue that it is important to pay more attention to GI manifestations and consider more careful examination when treating DM patients.

## Conflict of interest statement

The researchers claim for no conflicts of interests.

## Sources of funding

The author received no specific funding for this study.

## Ethical approval

This study was approved by the IRB in Jeju National University Hospital.

## Consent

Written informed consent was obtained from the patient's children for publication of this case report and accompanying images. A copy of the written consent is available for review by the Editor-in-Chief of this journal on request.

## Author contribution

Donghyoun Lee & Wooseong Jeong wrote the manuscripts. Chang Hak Hyun confirmed pathologic reports. Jinseok Kim reviewed the literature.

## Registration of research studies

NA.

## Guarantor

Jinseok Kim M.D., Ph.D.

## Data sharing statement

All additional data are available.

## Provenance and peer review

Not commissioned, externally peer-reviewed.
